# Data supporting consolidating emission indices of a diesel engine powered by carbon nanoparticle-doped diesel/biodiesel emulsion fuels using life cycle assessment framework

**DOI:** 10.1016/j.dib.2020.105428

**Published:** 2020-03-21

**Authors:** Homa Hosseinzadeh-Bandbafha, Meisam Tabatabaei, Mortaza Aghbashlo, Majid Khanali, Esmail Khalife, Taha Roodbar Shojaei, Pouya Mohammadi

**Affiliations:** a Department of Mechanical Engineering of Agricultural Machinery, Faculty of Agricultural Engineering and Technology, College of Agriculture and Natural Resources, University of Tehran, Karaj, Iran; b Faculty of Plantation and Agrotechnology, Universiti Teknologi MARA (UiTM), 40450 Shah Alam, Selangor, Malaysia; c Biofuel Research Team (BRTeam), Karaj, Iran; d Microbial Biotechnology Department, Agricultural Biotechnology Research Institute of Iran (ABRII), Agricultural Research, Education, and Extension Organization (AREEO), Karaj, Iran; e Faculty of Mechanical Engineering, Ho Chi Minh City University of Transport, Ho Chi Minh City, Vietnam; f Mechanical and Mechatronics Engineering Department, College of Engineering, Salahaddin University-Erbil, Erbil, Iraq

**Keywords:** Biodiesel, Emulsion fuel, Carbon nanoparticles, Diesel engine, Life cycle assessment

## Abstract

Integrated environmental analysis using life cycle assessment for different fuel blends used in a single-cylinder diesel engine was performed to select the most eco-friendly fuel blend. More specifically, the inventory data in support of the integrated environmental analysis of water-emulsified 5% biodiesel/diesel blends (B5) containing different levels of carbon nanoparticles (*i.e.*, 38, 75, and 150 µM) as a novel fuel nanoadditives at a fixed engine speed of 1000 rpm and four different engine loads (*i.e*., 25, 50, 75, and 100%) are presented. Neat diesel, B5, and B5 containing water (3 wt.%) were used as controls. Raw data related to the production and combustion of fuel blends were experimentally collected. Industrial (*i.e.*, experiments at large scale) and laboratory (*i.e.,* experiments at small scale) data were used for fuel blends production while experimental data obtained by engine tests were used for the combustion stage. Then raw data were processed with the IMPACT 2002+ methods by using the SimaPro software and EcoInvent database and were then converted into environmental impacts. Accordingly, six supplementary files including the inventory data on integrated environmental analysis of the different fuel blends are presented (Supplementary Files 1–6). The data could be applied for integrated environmental analysis in order to avoid subjective weighting of combustion parameters for selecting the most eco-friendly fuel blend for use in diesel engines. More specifically, by developing a single score indicator obtained through conducting integrated combustion analysis, comparison of various fuel blends is largely facilitated.

Specifications table

**Table:** 

Subject	Renewable Energy, Sustainability and the Environment
Specific subject area	Integrated combustion analysis by life cycle assessment
Type of data	Table and Figure
How data were acquired	Raw data related to the production to combustion of different fuel blends are collected. Industrial and laboratory data were used for fuel blends production while experimental data obtained by engine tests were used for the combustion stage. Then raw data are processed with the “IMPACT 2002+” methods using SimaPro software and EcoInvent database v3.0 and are converted into environmental impacts.
Data format	Raw and processed
Parameters for data collection	Energy and martial flow as well as emissions to air, water, and soil during fuel blends production. Power, torque, fuel and air consumed, *λ* (lambda) and exhaust gasses including CO_2_, CO, O_2_, and NO_x_ emissions during fuel blends combustion.
Description of data collection	Foreground data were collated directly during fuel blends preparation and engine tests. Background data were collated from the Ecoinvent v.3.0 database by using the SimaPro software.
Data source location	Tehran/ Iran
Data accessibility	Repository name: Mendeley Data; DOI: 10.17632/s73yrcp4v3.1 URL: https://data.mendeley.com/datasets/s73yrcp4v3/1 (http://dx.doi.org/10.17632/s73yrcp4v3.1)
Related research article	H. Hosseinzadeh-Bandbafha, M. Tabatabaei, M. Aghbashlo, M. Khanali, E. Khalife, T. Roodbar Shojaei, P. Mohammadi. Consolidating emission indices of a diesel engine powered by carbon nanoparticle-doped diesel/biodiesel emulsion fuels using life cycle assessment framework. Fuel, 267, 2020, 117,296. https://doi.org/10.1016/j.fuel.2020.117296

## Value of the data

• These data include the raw data required for conducting the LCA analysis of production and combustion of diesel, biodiesel, and carbon nanoparticle-doped diesel/biodiesel emulsion fuels. They could be used by future studies and save plenty of time and effort to build the required inventories.
• These data include the LCA processed data (related to the production and combustion of diesel, biodiesel, and carbon nanoparticle-doped diesel/biodiesel emulsion fuels), which could be used by future studies as a basis for comparing various fuel formulations.
• The data could be applied for integrated environmental analysis and to avoid subjective weighting of combustion parameters. It should be noted that more accurate decisions could be made on the environmental performance of fuel formulations (through the combustion process) using integrated environmental analysis.
• The data could be used for researchers and enthusiasts in the field of combustion, environment, material science, and chemical engineering for investigating the relationship between the environmental impacts of the shaft work obtained and combustion parameters of biodiesel blends containing water and carbon nanoparticles in comparison with neat diesel, B5, and B5 containing water.
• The data and the related platform introduced could be of use for researchers, fuel producers, and transportation managers to select the most eco-friendly fuel blend for diesel engines using a single score indicator obtained through conducting integrated environmental analysis.
• The data reveal that waste-oriented carbon nanoparticles investigated herein could serve as a promising eco-friendly fuel additives to enhance the environmental aspects of biodiesel/diesel blends. Moreover, the investigated additive could be regarded as a safer alternative for metal-based nanoadditives.

## 1 Data

This paper presents the data supporting the integrated environmental analysis of water-emulsified B5 containing three levels of carbon nanoparticles (CNPs) *i.e.*, 38, 75, and 150 µM (hereafter referred to as B5W3_CNP38_, B5W3_CNP75_, and B5W3_CNP150_, respectively) using life cycle assessment (LCA). While integrated environmental analysis of neat diesel, B5, and B5 containing 3 wt.% water (*i.e.* B5W3) are investigated as controls. These data present supplementary information to our recently published paper [1].

**Fig. 1:**
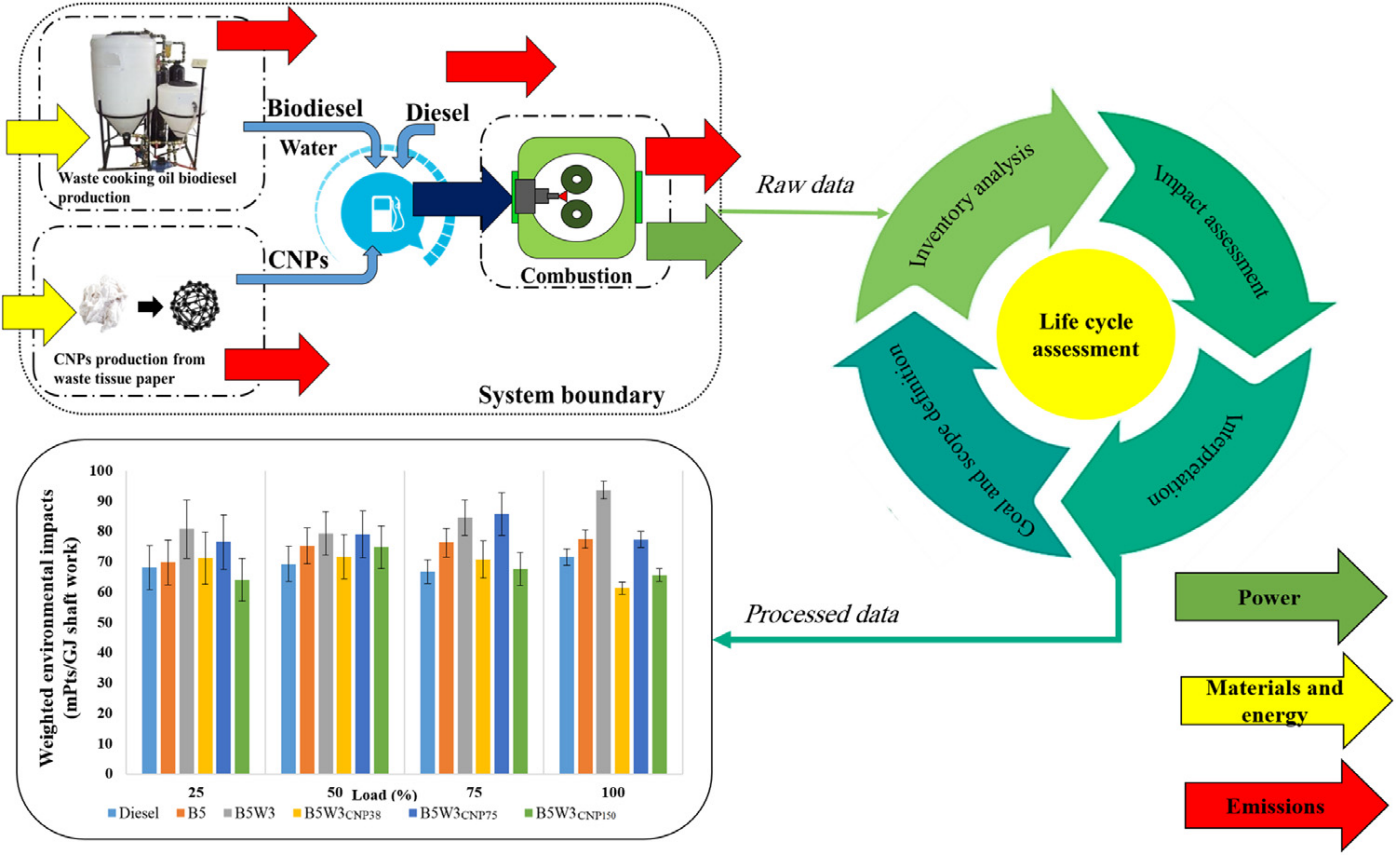
Summary of the conversion of raw data into processed data by LCA approch.

**Fig. 2:**
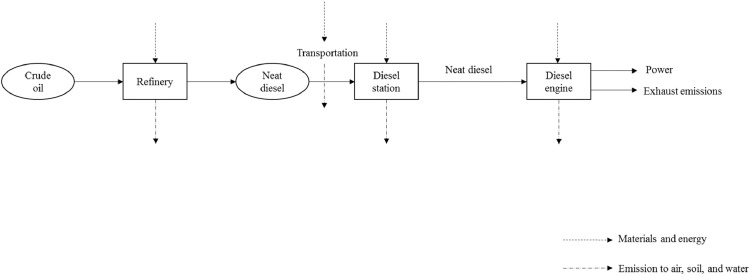
System boundary including the main steps involved in integrated environmental analysis of neat diesel.

**Fig. 3:**
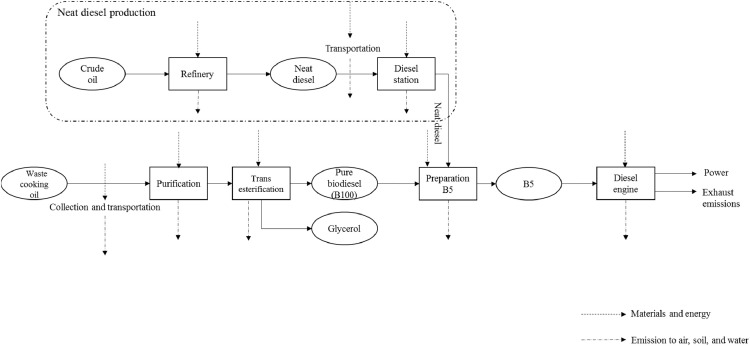
System boundary including the main steps involved in integrated environmental analysis of B5.

**Fig. 4:**
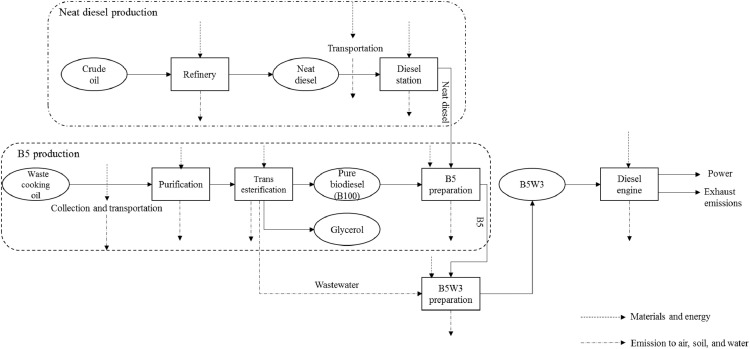
System boundary including the main steps involved in integrated environmental analysis of B5W3.

**Fig. 5:**
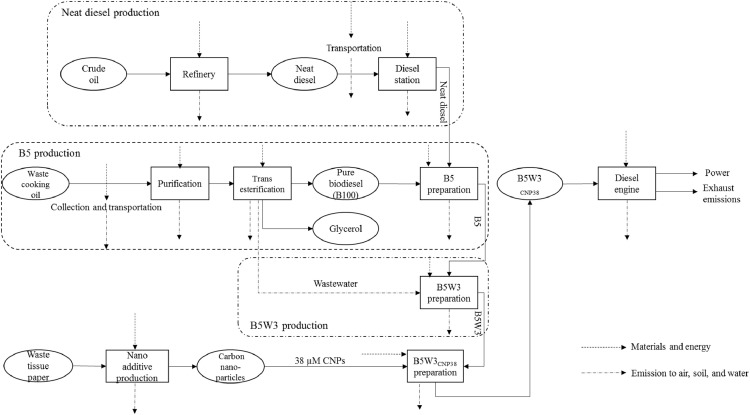
System boundary including the main steps involved in integrated environmental analysis of B5W3_CNP38_.

**Fig. 6:**
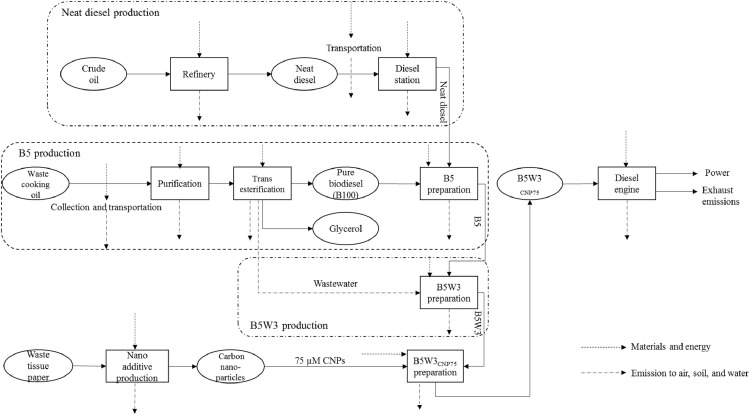
System boundary including the main steps involved in integrated environmental analysis of B5W3_CNP75_.

Based on the defined system boundaries (Figs 1 to 6), materials and energy employed for each GJ shaft work (well-to-shaft (WtS)) as well as for each kg of fuel blend (well-to-tank (WtT)) produced for each fuel blend and under different engine operating conditions were collected and imported into SimaPro software. In order to find the elementary flow to and from the environment associated with the materials and energy employed, the EcoInvent database v3.0 was applied. Details of the inventory data for six different fuel blends are defined in this database and are presented in the “Supplementary files 1 to 6” at https://data.mendeley.com/datasets/s73yrcp4v3/1 (http://dx.doi.org/10.17632/s73yrcp4v3.1). Supplementary files are named as follows:

• Supplementary file 1; Neat Diesel, Inventory Data
• Supplementary file 2; B5; Inventory Data
• Supplementary file 3; B5W3, Inventory Data
• Supplementary file 4; B5W3CNP38, Inventory Data
• Supplementary file 5; B5W3CNP75, Inventory Data
• Supplementary file 6; B5W3CNP150, Inventory Data

## 2 Experimental design, materials, and methods

Biodiesel as a stable, biodegradable, sulfur-free, and nontoxic fuel is a feasible alternative to mitigate the unfavorable environmental consequences associated with mineral diesel combustion [2,3]. However, there are still gaps and limitations to be addressed in order to consider biodiesel as an ideal fuel [4,5]. For instance, lower calorific value and higher nitrogen oxides (NO_x_) emissions of biodiesel and its blends compared with diesel are among such shortcomings [6,7]. Currently, application of fuel additives to overcome these challenges is one of the most acceptable approaches [8]. Among different fuel additives, water has been found promising [9], but its presence in fuel can also lead to problems such as prolonged ignition delay [10,11]. For solving this problem, metallic/non-metallic nanoadditives have been employed [12], [13], [14]. Nevertheless, there exist some limitations/concerns associated with the application of metallic nanoadditives such as post-combustion faith of the nanomaterials residues. Non-metallic nanoadditives (*e.g.,* various forms of carbon nanomaterials) are also attributed with a number of unfavorable features such as fuel instability (sedimentation) and alteration in fuel color. To address the above-mentioned limitations, the application of aqueous solution of waste-oriented CNPs as safe and stable fuel nanoadditive was investigated herein.

Unlike the existing literature in which discrete conventional analysis is used to assess the combustion parameters of different fuel blends, the present method took advantage of an integrated LCA-based approach, *i.e.,* integrated environmental analysis to assess the environmental impacts of different fuel blends investigated. In better words, integrated environmental analysis is an accurate non-subjective weighting performed based on the LCA approach and is capable of estimating the favorable and/or unfavorable impacts of power generation by various fuel blends throughout the various phases of their life cycle. More specifically, these data demonstrate how raw data related to fuel production and combustion (*i.e.*, engine performance and exhaust emissions data) could be integrated by LCA damage categories (including human health, ecosystem quality, climate change, and resources damage categories) to accurate non-subjective single score data (Fig. 1). Moreover, these data could also facilitate future research works on the LCA of production and combustion of diesel, biodiesel, and carbon nanoparticle-doped diesel/biodiesel emulsion fuels. Such data could save plenty of time and effort required to build the inventories.

According to ISO 14,040 and 14,044, LCA has four phases including: (1) goal and scope definition, (2) inventory analysis, (3) impact assessment, and (4) interpretation [15]:

### 2.1 Goal and scope definition

The main goal of this work was to obtain and present the integrated environmental analysis inventory data for the production of the investigated fuel blends followed by their combustion at a fixed engine speed of 1000 rpm and at four different engine loads (*i.e.,* 25, 50, 75, and 100%). The inventory data were obtained based on a specific function unit (FU) and defined system boundary.

Integrated environmental analysis was performed in two general system boundaries: WtT, *i.e.,* fuel blends production and WtS, *i.e.,* production to combustion of the fuel blends. Inventory data to and from the system boundary were collected and calculated based on 1 kg fuel blend and 1 GJ shaft work to estimate the environmental impacts of WtT and WtS, respectively. On such basis, the system boundary for the shaft work obtained by six different fuel blends were defined in this database, including neat diesel, B5, B5W3, B5W3 containing different of carbon nanoparticles, *i.e.*, 38, 75, and 150 µM:

*Neat diesel:* This system boundary is based on the shaft work obtained through neat diesel combustion in a single cylinder diesel engine. As presented in Fig. 2, “crude oil” is converted into neat diesel in oil refineries and is then transmitted to diesel stations. The data related to the extraction and conversion of crude oil into neat diesel was taken from the EcoInvent database v3.0. Subsequently, the fuel is combusted in a 0.507 L single-cylinder, water-cooled, four-stroke, and variable compression ratio (VCR) (Max. CR 22) direct injection Ricardo E6 diesel engine coupled with a 22-kW electrical dynamometer for loading. It should be mentioned that engine was warmed up and the measuring equipment were calibrated prior to the combustion of the fuel blends at a fixed engine speed of 1000 rpm and four different engine loads (*i.e.*, 25, 50, 75, and 100%). Fuel consumption and Air Fuel Ratio (AFR) were measured by an AVL-735 fuel mass flowmeter and a Lambda sensor, respectively. In addition, an infrared-based AVL gas analyzer was used to measure carbon dioxide (CO_2_), carbon monoxide (CO), and unburned hydrocarbons (HC) emissions. Finally, the amount of oxygen (O_2_) and NO_x_ emissions were determined by electrochemical method. The data collected from the engine tests for neat diesel are listed in Supplementary file 1; Neat Diesel, Inventory Data/Raw data/Data measured during combustion.xlsx.

*B5; 5% blend of waste cooking oil (WCO) biodiesel and neat diesel:* this system boundary is based on the shaft work obtained through the combustion of neat diesel containing 5 wt.% WCO biodiesel. System boundary includes five main steps (Fig. 3) including: collection and transportation of WCO, oil refining, production of biodiesel (transesterification), preparation of B5, and the combustion of B5 in the diesel engine. After a pre-treatment step using sulfuric acid, the free fatty acids content of WCO was decrease decreased and then, the pretreated oil was converted into methyl esters through the transesterification reaction with methanol and in the presence potassium hydroxide (KOH) as catalyst in a stirred tank reactor for 1 h at 60 °C. Following glycerol separation by gravity and the removal of methanol–water, the remaining methyl esters were washed with water three times using the method previously described by Jaber et al. [16]. Then 5 wt.% WCO biodiesel was mixed with 95 wt.% diesel and was labeled as B5. The raw data collected for the transesterification of WCO and B5 preparation are listed in Supplementary file 2; B5, Inventory Data/Raw data/Data for B100 production.xlsx and Supplementary file 2; B5, Inventory Data/Raw data/Data for B5 preparation.xlsx, respectively. Engine specifications and experimental procedure were the same as those used for neat diesel. The data related to B5 preparation and engine tests are listed in Supplementary file 2; B5, Inventory Data/Raw data/Data measured during combustion.xlsx.

*B5W3; 3* *wt.% water inclusion into B5:* This system boundary is based on the shaft work from the combustion of B5 containing 3 wt.% water. B5 preparation, blending water with B5 (emulsification), and combustion of the fuel blend are the major steps (Fig. 4). Emulsification of water in diesel/biodiesel blend was accomplished by using 75 mL of a 1:2 blend of Polysorbate 80 and Sorbitan monooleate (Merck, Germany) prior to the addition of water. It should be noted that to stabilize the prepared emulsions, a Polytron® homogenizer (Switzerland) was used at room temperature for 15 min (see Supplementary file 3; B5W3, Inventory Data/Raw data/Data for B5W3 preparation.xlsx). Engine specifications and experimental procedure were the same as those used for neat diesel and B5. The data related to B5W3 preparation and engine tests are listed in Supplementary file 3; B5W3, Inventory Data/Raw data/Data measured during combustion.xlsx.

B5W3_CNP38_; *38* *µM CNPs inclusion into B5W3*: this system boundary is based on the shaft work obtained through the combustion of B5W3 containing 38 µM CNPs in the diesel engine (Fig. 5). The only difference between this fuel blend and the B5W3 is presence of CNPs (spherical and approx. 45 nm in size) as carbon-based nanoadditives in the fuel blend. Notably, CNPs are classified as a remarkable type of quasispherical carbonaceous nanomaterials and due to the existence of numerous carboxylic moieties on their surface, are highly solubile in water [17]. There are four main steps in this system boundary including: (1) CNPs production from waste tissue paper (briefly, after washing by water, waste tissue papers were dried in an oven for 2 h at 37 °C. Subsequently, under atmospheric air, waste tissue papers were entirely burned. The remaining ash form the burned waste tissue papers was diffused in 46.7 mL double distilled water. Then, 3.3 mL of nitric acid was added to the obtained suspension and the mixture was stirred for 30 min and was subsequently sonicated for 60 min. At this stage, the suspension was filtered through a filter paper and was then centrifuged at 7000 × *g* for 15 min. Eventually, a transparent solution was obtained by dialysis against double distilled water to remove residual impurities; See Supplementary file 4; B5W3_CNP38_, Inventory Data/Raw data/Data for CNP production.xlsx.), (2) B5 preparation, (3) water-emulsification and CNPs addition into B5 (See Supplementary file 4; B5W3_CNP38_, Inventory Data/Raw data/Data for B5W3_CNP38_ preparation.xlsx), and finally, (4) combustion of B5W3_CNP38_. Engine specifications and experimental procedure were the same as the above-mentioned fuel blends. The data related to B5W3_CNP38_ preparation and engine tests are listed in Supplementary file 4; B5W3_cnp38_, Inventory Data/Raw data/Data measured during combustion.xlsx.

B5W3_CNP75_; *75* *µM CNPs inclusion into B5W3*: This system boundary is based on the shaft work obtained through the combustion of B5W3_CNP75_ (Fig. 6). The only difference between this fuel blend and B5W3_CNP38_ is the concentration of CNPs. Engine specifications and experimental procedure were the same as the above-mentioned fuel blends. The data related to this fuel blend are listed in Supplementary file 5; B5W3_cnp75_, Inventory Data.xlsx.

**Fig. 7:**
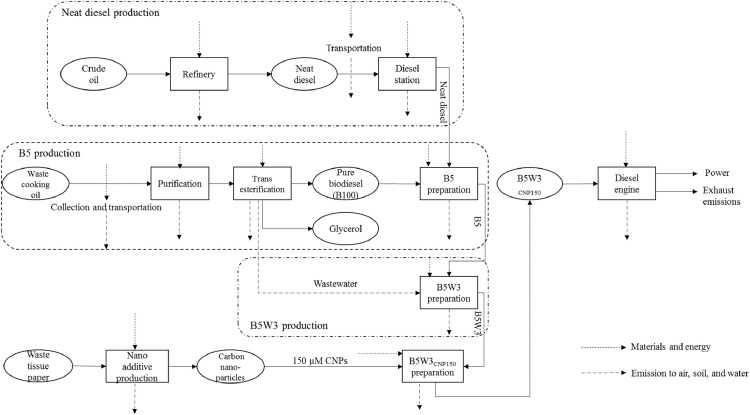
System boundary including the main steps involved in integrated environmental analysis of B5W3_CNP150_.

B5W3_CNP150_; *150* *µM CNPs inclusion into B5W3:* This system boundary is based in the shaft work obtained through the combustion of B5W3_CNP150_ (Fig. 7). The only difference between this fuel blend compared with B5W3_CNP38_ and B5W3_CNP75_ is CNPs concentration in the fuel blend. Engine specifications and experimental procedure were the same as the above-mentioned fuel blends. Supplementary file 6; B5W3_cnp150_, Inventory Data.xlsx is related to this fuel blend.

### 2.2 Life cycle inventory

Life cycle inventory (LCI) analysis involves developing an inventory of flows of energy, materials, and emissions to air, soil, and water for a product based on a clear picture of the technical system boundary in the form of elementary flow to and from the environment [15]. The elementary flow to and from the environment for the fuel blends investigated in this database for the WtT and WtS approaches are shown in Supplementary files 1–6.

### 2.3 Life cycle impact assessment

The life cycle inventory is usually organized from more than 1000 elementary flows having various units. On the contrary, the purpose of the integrated environmental analysis is to integrate combustion parameters using the LCA approach to provide a non-subjective single indicator. This could consequently make possible the understanding and interpretation of the elementary flows of the shaft work generated.

In fact, integrated environmental analysis is accomplished through carrying out life cycle impact assessment (LCIA). More specifically, LCIA cumulates the inventory data based on elementary flows into a manageable number that helps to understand the environmental impact of a product or service [18]. LCIA includes different elements, such as: characterization, grouping, normalization and weighting [19]. In the characterization, elementary flows are combined and converted into indicators that reflect the environmental impacts of the process based on various impact categories such as, carcinogen, global warming, acidification, *etc.* (midpoints)*.* Impact categories are combined together and are assigned into a lower number of sets to better allow the interpretation such as, human health, ecosystem quality, climate change, and resources depletion (endpoints). Finally, in the weighting, elementary flows are weighted based on societal preferences [15]. Consequently, LCIA can convert the data obtained based on the elementary flows of the shaft work obtained into a single score, providing non-subjective outputs; *i.e.,* the main target of integrated combustion analysis. Different LCIA methods can be used while in this work, the inventory data were modeled based on the elementary flows by using IMPACT 2002 + method (*via* 15 midpoint categories and four damage categories [20]) available in SimaPro 8.2.3.0 (Supplementary files 1–6).

## Appendix Supplementary materials

Image, application 1

Image, application 2
