# Supplementary material for: Data supporting consolidating emission indices of a diesel engine powered by carbon nanoparticle-doped diesel/biodiesel emulsion fuels using life cycle assessment framework
Source: Data Brief. 2020 Mar 21;30:105428. doi: 10.1016/j.dib.2020.105428 (PMC7160430; doi:10.1016/j.dib.2020.105428)
Supplement: Supplementary file 2 [file mmc2.docx]

https://data.mendeley.com/datasets/s73yrcp4v3/1
